# Emergency Department Boarding, Inpatient Census, and Interhospital Transfer Acceptances

**DOI:** 10.1001/jamanetworkopen.2025.12299

**Published:** 2025-05-28

**Authors:** Margaret Greenwood-Ericksen, Neil Kamdar, Kjirsten Swenson, Peter Pruitt, Marta L. McCrum, Gabriel Paul, Larissa Myaskovsky, Keith E. Kocher, Kori S. Zachrison

**Affiliations:** 1 Department of Emergency Medicine, School of Medicine, University of New Mexico, Albuquerque; 2 Institute for Healthcare Policy and Innovation, University of Michigan, Ann Arbor; 3 Center for Population Health Sciences, Stanford University, Stanford, California; 4 Sheps Center for Health Services Research, University of North Carolina, Chapel Hill; 5 Department of Emergency Medicine, Northwestern University Feinberg School of Medicine, Chicago, Illinois; 6 Department of Surgery, University of Utah, Salt Lake City; 7 Department of Internal Medicine, University of New Mexico, School of Medicine, Albuquerque; 8 Departments of Emergency Medicine and Learning Health Sciences, University of Michigan, Ann Arbor; 9 Department of Emergency Medicine, Massachusetts General Hospital, Boston; 10 Department of Emergency Medicine, Harvard Medical School, Boston, Massachusetts

## Abstract

**Importance:**

Referral hospitals in the US are experiencing unprecedented levels of crowding, leading them to increasingly refuse interhospital transfer (IHT) requests. Crowded hospitals are dangerous, but refusing IHTs undermines the role of referral hospitals and may cause harm.

**Objective:**

To measure associations of hospital crowding measures (emergency department [ED] boarding and inpatient census) with IHT acceptances overall and for prioritized conditions.

**Design, Setting, and Participants:**

This cross-sectional study from January 2019 to May 2023 analyzed data from the only academic and level I trauma center in a highly rural state in the Southwestern US, including transfer center data, ED boarding hours, and inpatient census. All transfer center calls regarding adults (age >18 years) were eligible for the study. Data were analyzed from June to October 2024.

**Main Outcome and Measures:**

The primary outcome was the proportion of transfer requests accepted on a weekly and monthly basis. Adjusted logistic regression was used to analyze associations of ED boarding time and inpatient census with IHT acceptance, considering prioritized conditions (obstetrics, ST-elevation myocardial infarction [STEMI], stroke, and trauma) and rurality. Transfer data contained IHT request descriptors, including referring facility, date and time of call, decision (accept or decline), diagnosis, and patient demographics. ED boarding was measured daily as a sum of all boarding hours for each ED patient.

**Results:**

The study included 26 020 IHT requests (11 267 women [43.2%]; mean [SD] age, 54.4 [19.6] years), of which 16 062 were accepted (61.7%). There were 22 119 (85.0%) requests from urban and 3901 requests (15.0%) from rural hospitals, with the majority of IHT requests (19 912 requests [76.3%]) seeking transfer from an ED. There was a negative correlation between IHT acceptance and ED boarding (Pearson *r*, −0.73) and inpatient census (Pearson *r*, −0.87). At times of worst ED boarding (highest vs lowest quartile), the odds of IHT acceptance were lower (adjusted odds ratio [aOR], 0.71; 95% CI, 0.66-0.78). Of the 3901 rural requests, 2196 (56.3%) were accepted, with lower odds of acceptance for rural vs urban requests (aOR, 0.66; 95% CI, 0.64-0.79). Prioritized diagnoses were more commonly accepted, particularly obstetrics (aOR, 5.28; 95% CI, 4.17-6.70), STEMI (aOR, 3.04; 95% CI, 1.86-4.98), and trauma (aOR, 3.19; 95% CI, 2.86, 3.57).

**Conclusions and Relevance:**

In this cross-sectional study of IHT requests, the severity of ED boarding and inpatient census were associated with decreased IHT acceptance, suggesting that overcrowded referral hospitals face tradeoffs as they seek to fulfill seemingly conflicting obligations to safely care for locally hospitalized patients and accept regional patients seeking transfer.

## Key Points

**Question:**

Are emergency department (ED) boarding and inpatient census associated with interhospital transfer (IHT) acceptances?

**Findings:**

This cross-sectional study of 26 020 IHT requests found that the severity of ED boarding and inpatient census were associated with declining IHT acceptance.

**Meaning:**

These findings suggest that physicians at overcrowded referral hospitals face tradeoffs with implications for both rural and urban patients as they seek to fulfill seemingly conflicting obligations to safely care for locally hospitalized patients and accept regional patients seeking transfer.

## Teaser

This cross-sectional study analyzed associations of hospital crowding measures (emergency department [ED] boarding and inpatient census) with interhospital acceptances in the rural Southwestern US.

## Introduction

Across the US, the emergency departments (EDs) of referral hospitals are experiencing a crisis of ED boarding, where admitted patients remain in the ED while they await inpatient beds.^1^ The immediate cause is extreme inpatient census, driven by a greater demand on inpatient capacity, increasing length of stay for hospitalized patients, and difficulty accessing placement to outpatient services such as skilled nursing facilities.^2,3^ As hospital workflow becomes paralyzed, there are negative downstream impacts on the ED, which despite having a fixed number of treatment spaces, is often the only hospital unit expected to flex to patient surges. The result is higher states of ED boarding and associated crowded waiting rooms. Crowded EDs are dangerous and are associated with increased mortality and morbidity for both ED and hospitalized patients.^3,4,5^ Boarding and crowding conditions worsened during the COVID-19 pandemic due to extreme inpatient census.^6,7,8^ One of the tactics deployed by hospitals to address capacity challenges was reducing additional patient influx by strictly limiting acceptances of interhospital transfer (IHT) requests to the ED and inpatient setting. Reduced IHT acceptance was common during the COVID-19 pandemic and has persisted due to ongoing tertiary hospital capacity challenges.^8^ However, these approaches may result in harm to patients at outlying hospitals due to delay in care for time-sensitive conditions that rely exclusively on IHT to access life-saving services at tertiary care centers.^9,10^

Although ED boarding harms local patients, addressing this crisis by reducing IHT acceptances may cause harm to patients hospitalized at rural and suburban community hospitals within the same region. These patients rely on IHT to access specialized services and procedures only available at higher volume centers. Prior to the COVID-19 pandemic, rural ED patients experienced at least twice as many IHTs as urban patients, with 6% to 15% of ED visits resulting in transfer^11^ and up to 30% for some conditions.^12^ In effect, by limiting IHT acceptances, receiving hospitals are limiting access to such care for patients who seek care at outlying hospitals. This approach can potentially widen disparities in access to care. A recent study^8^ demonstrated that during the COVID-19 pandemic, there were paradoxical decreases in overall patient transfer during periods of high census. Our study builds on these findings, measuring the association of ED boarding and inpatient capacity with IHT acceptance, and exploring the differential impact on geographic location and by diagnosis.

We tested the hypothesis that increased levels of referral hospital ED boarding would be associated with a decreased rate of IHT acceptances, disproportionately affecting rural patients. Because inpatient census is less elastic than ED census, we also examined the association of inpatient census with IHT acceptance, given the interconnectedness between ED and inpatient capacity. We examined if prioritization of conditions (eg, stroke or acute coronary syndrome) has a differential impact for urban vs rural patients. We used data from an ideal setting, leveraging transfer information from the only academic medical center serving a highly rural state.

## Methods

### Setting

This cross-sectional study included data from a single institution and its transfer center in a highly rural state in the Southwestern US, serving a highly racially and ethnically diverse population (13% Native American and 52% Hispanic).^13^ The hospital is licensed for 308 adult inpatient beds and 72 intensive care beds, and the adult ED (excluding urgent care) sees an annual volume of 75 036 patient encounters with an average admission rate of 29%. It is the only level I trauma, comprehensive stroke, and high-risk obstetrics center within the state. As such, they never decline IHT requests for obstetrics, multisystem trauma, cerebrovascular event (stroke), and ST-elevation myocardial infarction (STEMI). The hospital houses a statewide consult and transfer center, which receives more than 30 000 calls a year. The transfer center has routine processes for data collection (eg, date and time of call, location, and diagnosis) during transfer requests. The Southwestern state experienced 2 surges of COVID-19 in spring 2020 and fall 2021. As community need expanded, the hospital was permitted to expand beyond licensed capacity in spring 2021, which resulted in a rapid increase inpatient census. Concurrently, to preserve access for patients with conditions for which this academic medical center is the only treatment center (eg, endovascular stroke or high-risk obstetrics), the hospital sought to reduce transfers of conditions that could be safely cared for at other community hospitals (eg, sepsis or respiratory failure); this became an informal policy, which prioritized acceptance of trauma, STEMI, stroke, obstetrics, and pediatrics. This study was approved by The University of New Mexico Health Science Center institutional review board and was exempt from informed consent due to the use of deidentified administrative data. The reporting follows the Strengthening the Reporting of Observational Studies in Epidemiology (STROBE) reporting guideline for cross-sectional studies.

### Population

**Figure 1.**
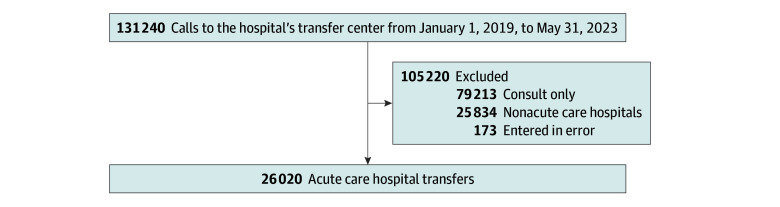
Study Population Inclusion and Exclusion Criteria Nonacute care hospital is defined as transfers originating from an entity that is not credentialed as an acute care hospital and includes outpatient clinics or surgical centers, skilled nursing facilities, and other residential facilities (eg, nursing homes).

All transfer center calls from January 2019 to May 2023 regarding adults (age >18 years) were eligible for the study (Figure 1). From this sample, we applied several exclusion criteria. First, we excluded calls for consult only, which is a service offered by the transfer center. In the case that a consult call evolves from a consult to a transfer request, they are reclassified as a transfer request and are thus captured in the dataset as a transfer. Second, we restricted our study population to transfer requests from acute care hospitals (as opposed to clinics or nursing homes, for example) because these are always accepted regardless of capacity constraints. Third, there were several error entries, which we removed. Our rationale for excluding children (age <18 years) is that these represent a different care delivery model.

### Datasets and Variables

Transfer center data contains IHT request descriptors, collected in a standardized way for all transfer request calls. The data elements include referring facility, patient location at referring hospital (eg, ED, intensive care unit [ICU], or floor), time the call is received, specialty service requested, requested level of care (ED, ICU, or floor), patient demographics (age and gender), decision (accept or decline), and time the decision is made on the request. Diagnosis categories were also collected, selected from a drop-down menu, and reflect the presumed diagnosis at time of IHT request. The decision to accept or decline an IHT request is made collaboratively between an ED physician working in the transfer center, specialist (if involved), and sending clinician. Rurality was determined by referring hospital zipcode and categorized by rural-urban commuting areas codes.^14^

We linked transfer center data to daily ED boarding hour data from the ED. This variable is measured daily at midnight for quality improvement purposes. It is defined as the sum of all boarding hours for each admitted patient who remains in the ED. Boarding hours for each patient are calculated starting from the date and time order for a bed request, and ending either at midnight (if still in ED) or at time of departure from ED. The sum across patients in the department during the previous 24 hours generates the daily value for ED boarding hours.

### Statistical Analysis

The primary outcome of interest was the proportion of transfer requests accepted (accepted divided by all requests) on a weekly and monthly basis. Descriptive statistics were calculated for transfer requests, comparing accepted vs declined requests and rural vs urban requests. The proportion of accepted requests was calculated on a weekly basis and compared graphically with weekly ED boarding hour averages (means). Because inpatient census is a presumed upstream factor associated with boarding, we also were interested in the association of inpatient census with IHT acceptance. However, inpatient (excluding ICU) census was only available as a monthly average, so we used monthly ED boarding hour averages (means) for comparison. We identified the top 20 diagnoses for accepted transfer requests and noted those prioritized (eTable 1 in Supplement 1). We first determined unadjusted associations of ED boarding with IHT request acceptances. We then used a logistic regression model to analyze the association of ED boarding time with rate of acceptance by week, adjusting for age, gender, weekend, referring unit, rurality, and diagnosis category. In this model, ED boarding hours were categorized by quartile. Statistical analysis was conducted from June to October 2024 using SAS software version 9.4 (SAS Institute). The threshold for statistical significance was a 2-sided *P *< .05.

We conducted 2 sensitivity analyses with interaction terms examining whether the association of boarding with acceptance of IHT requests varied for individual conditions prioritized by the transfer center policy (STEMI, obstetrics, trauma, or stroke). First, our experience at the transfer center led us to hypothesize that rural requests would be more commonly declined, given they must transfer patients with lower acuity due to lack of resources. However, we also hypothesized that for high-acuity conditions, urban and rural transfers would be equally likely to be accepted. Thus, we compared prioritized high-acuity diagnoses (STEMI, obstetrics, trauma, or stroke) vs all other requests. We examined the adjusted proportion of acceptances between urban vs rural requests and report odds ratios (ORs) and estimated means.

Second, to explore ED-only comparisons, we limited the sample to ED-originating IHT requests. Similarly, we examined the acceptance proportion overall and by prioritized conditions, using our adjusted model, and report ORs and estimated means. For both sensitivity analyses, ORs are the adjusted odds of acceptance within diagnosis category between rural vs urban requests, with urban as the referent. For both analyses, the estimated means compare the odds of a prioritized condition (eg, obstetrics, STEMI, stroke, or trauma) being accepted from an urban and rural hospital. Estimated means are based on the least square means estimates and are the estimated population margins. These represent the estimates of the marginal means over a balanced population.

## Results

Our study sample included 26 020 IHT requests (11 267 women [43.2%]; mean [SD] age, 54.4 [19.6] years), of which 16 062 (61.7%) were accepted. There were 22 119 requests (85.0%) originating from urban hospitals and 3901 requests (15.0%) from rural hospitals. The majority of IHT requests (19 912 requests [76.3%]) originated from the ED (Table 1). For IHT requests, those from urban vs rural hospitals were more commonly accepted (13 866 requests [62.5%] vs 2196 requests [56.3%]), although when comparing urban vs rural ED IHT requests, those from rural EDs were proportionally more commonly accepted (1892 requests [86.2%] vs 10 862 requests [78.3%]) (Table 2).

**Table 1.** Characteristics of Interhospital Transfer Requests (Urban vs Rural)

Characteristic	Interhospital transfer requests, No. (%)		
	All (N = 26 020)	Urban (n = 22 119)	Rural (n = 3901)
Age, mean (SD), y	54.4 (19.6)	54.4 (19.6)	52.6 (19.2)
Gender			
Man	14 753 (56.8)	12 541 (56.7)	2247 (57.6)
Woman	11 267 (43.2)	9, 577 (43.3)	1654 (42.4)
Referring unit seeking treatment			
Emergency department	19 912 (76.3)	16 639 (75.0)	3273 (83.9)
Floor	4564 (17.5)	4092 (18.4)	472 (12.1)
Intensive care unit	1624 (6.2)	1468 (6.6)	156 (4.0)
Emergency department boarding, mean (SD), h	603.2 (329.9)	601.2 (326.1)	614.23(337.6)
Weekend request	7566 (29.0)	6459 (29.2)	1077 (27.6)

**Table 2.** Characteristics of Urban and Rural Transfer Requests (Accepted vs Declined)

Characteristic	Transfer requests, No./total No. (%)		
	All transfer requests	Requests accepted	Requests declined
Urban transfer requests			
Age, mean (SD), y	54.4 (19.6)	52.9 (19.7)	56.8 (19.1)
Gender			
Woman	9613/22 119 (43.5)	5947/13 866 (42.9)	3666/8333 (44.0)
Man	12 506/22 119 (56.5)	7919/13 866 (57.1)	4667/8333 (56.0)
Referring unit seeking treatment			
Emergency department	16 639/22 119 (75.0)	10 862/13 866 (78.3)	5777/8333 (69.3)
Floor	4092/22 119 (18.4)	2221/13 866 (16.0)	1871/8333 (22.5)
Intensive care unit	1468/22 119 (6.6%)	783/13 866 (5.7)	685/8333 (8.2)
Emergency department boarding, mean (SD), h	601.2 (326.1)	552.0 (302.7)	684.1 (347.0)
Weekend request	6488/22 119 (29.2)	4231/13 866 (30.5)	2257/8333 (27.1)
Rural transfer requests			
Age, mean (SD), y	52.6 (19.2)	50.2 (19.1)	55.6 (18.8)
Gender			
Woman	1653/3901 (42.4)	921/2196 (41.9)	732/1705 (42.9)
Man	2248/3901 (57.6)	1275/2196 (58.1)	973/1705 (57.1)
Referring unit seeking treatment			
Emergency department	3273/3901 (83.9)	1892/2196 (86.2)	1381/1705 (81.0)
Floor	472/3901 (12.1)	214/2196 (9.7)	258/1705 (15.1)
Intensive care unit	156/3901 (4.0)	90/2196 (4.1)	66/1705 (3.9)
Emergency department boarding, mean (SD), h	614.3 (337.6)	541.1 (308.1)	710.5 (350.5)
Weekend request	1077/3901 (27.6)	650/2196 (29.6)	427/1705 (25.0)

**Figure 2.**
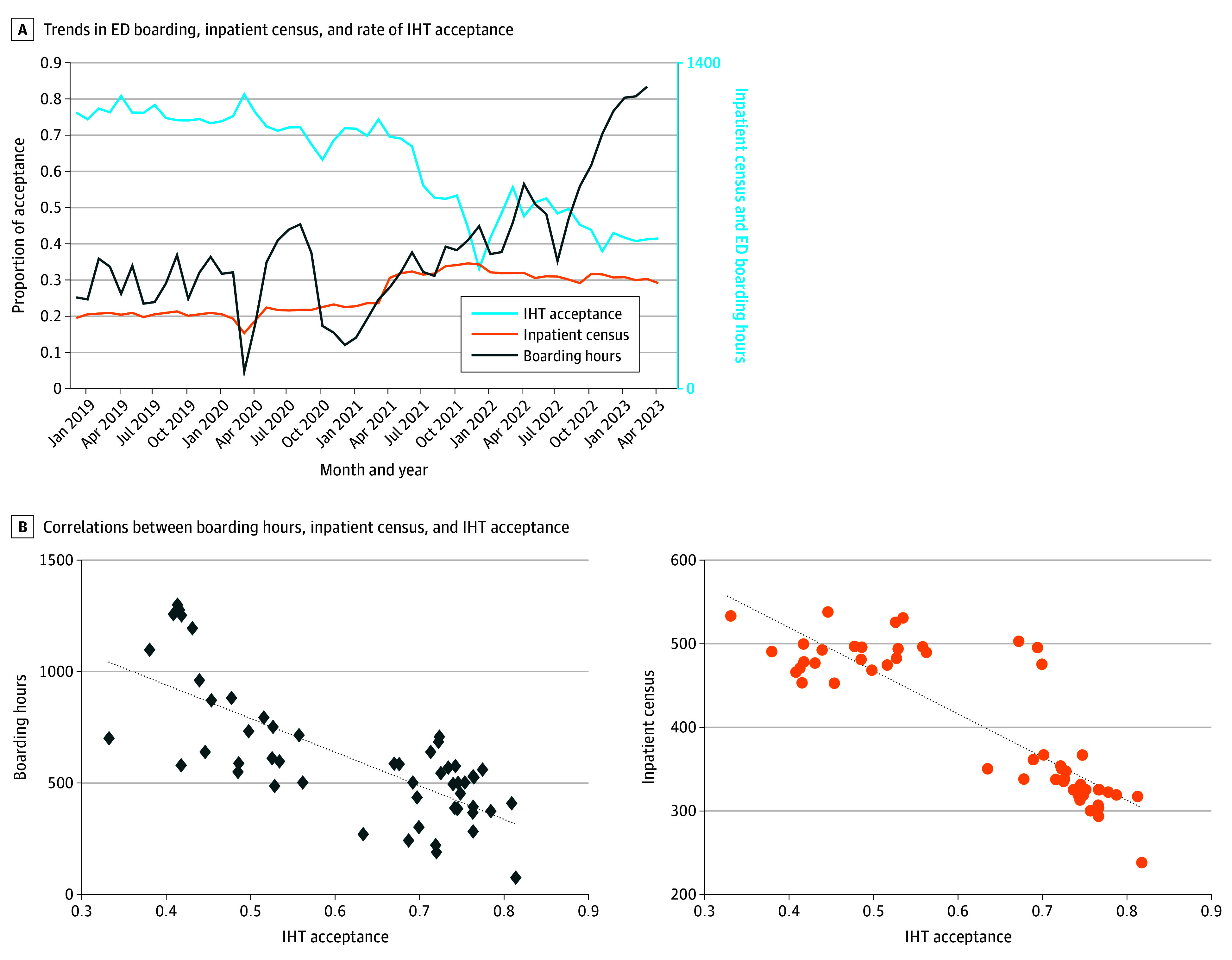
Emergency Department (ED) Boarding, Inpatient Census, and Rate of Interhospital Transfer (IHT) Acceptance by Month ED boarding and inpatient census are measured as a monthly average (mean). The acceptance proportion is calculated by accepted transfer requests and total transfer requests by month. In March to April 2020, the state’s Department of Health (DOH) messaged avoiding EDs and health care for lower acuity concerns, the timing of which was associated with a decrease in ED volume and inpatient admissions; this coincided with the first surge of COVID-19, which occurred primarily in the state’s tribal regions, resulting in many transfers from the Navajo Nation and Pueblo communities to the academic medical center. In March 2021, to meet community needs and expand capacity, the hospital and the state DOH petitioned the Centers for Medicare and Medicaid to expand past the hospital’s licensed bed capacity, and this was granted in the spring of 2021. In September 2021, the second surge was in metro regions. In fall 2021, the expanded capacity allowed for greater hospitalizations. In summer 2022, there was a gradual reduction in inpatient volume, reducing inpatient crowding, and a rise in ED boarding. In the summer of 2023, which is not included, the hospital returned to licensed bed capacity (per DOH order).

The hospital maintained an IHT acceptance rate of more than 70% prior to the COVID-19 pandemic, which plummeted to 33% in January 2022, a decrease which coincides with peaking inpatient census (mean monthly inpatient census, 533.5 patients) (eTable 2 in Supplement 1). The IHT acceptance rate plateaued at approximately 40%, while a slow decline in inpatient census preceded a rapid rise in ED boarding in summer 2022 (Figure 2A). We found greater monthly ED boarding and inpatient census were highly negatively correlated with lower transfer acceptance (Pearson *r*, −0.73 and −0.87, respectively) (Figure 2B).

In our adjusted model (Table 3), overall higher quartiles, compared with the first quartile of ED boarding, were associated with lower odds of acceptance (fourth quartile [highest boarding]: adjusted OR [aOR], 0.71; 95% CI, 0.66-0.78). In addition, all individual prioritized diagnoses, including obstetrics (aOR, 5.28; 95% CI, 4.17-6.70), STEMI (aOR, 3.04; 95% CI, 1.86-4.98), stroke (aOR, 1.65; 95% CI, 1.41-1.94), and trauma (aOR, 3.19; 95% CI, 2.86, 3.57), had greater odds of acceptance as compared with all other diagnoses (Table 3). Rural IHT requests had lower odds of acceptance than urban requests (aOR, 0.66; 95% CI, 0.52-0.82). ED transfers had greater odds of acceptance than ICU (aOR, 0.71; 95% CI, 0.64-0.79) or floor (aOR, 0.61; 95% CI, 0.56-0.65) transfers, and all transfers were more likely to be accepted on a weekend vs a weekday (aOR, 1.08; 95% CI, 1.01-1.16).

**Table 3.** Factors Associated With Interhospital Transfer Acceptance in Unadjusted and Adjusted Analyses^a^

Factor	Unadjusted OR (95% CI)	*P* value	Adjusted OR (95% CI)	*P* value
Gender				
Man	1 [Reference]	NA	1 [Reference]	NA
Woman	1.05 (0.99-1.1)	.98	1.00 (0.95-1.06)	.92
Boarding hours, quartile				
1	1 [Reference]	NA	1 [Reference]	NA
2	0.70 (0.65-0.76)	<.001	0.75 (0.69-0.82)	<.001
3	0.63 (0.58-0.67)	<.001	0.71 (0.66-0.77)	<.001
4 (ie, Highest boarding)	0.37 (0.35-0.40)	<.001	0.71 (0.66-0.78)	<.001
Rural vs urban				
Urban	1 [Reference]	NA	1 [Reference]	NA
Rural	0.77 (0.72-0.83)	<.001	0.66 (0.52-0.82)	<.001
Referring unit				
Emergency department	1 [Reference]	NA	1 [Reference]	NA
Floor	0.64 (0.60-0.68)	<.001	0.61 (0.56-0.65)	<.001
Intensive care unit	0.65 (0.59-0.72)	<.001	0.71 (0.64-0.79)	<.001
Weekend vs weekday request				
Weekday	1 [Reference]	NA	1 [Reference]	NA
Weekend	1.19 (1.13-1.26)	<.001	1.08 (1.01-1.16)	<.001
Diagnosis category				
All others	1 [Reference]	NA	1 [Reference]	NA
Obstetrics	4.3 (3.56-5.16)	<.001	5.28 (4.17-6.70)	<.001
ST-elevation myocardial infarction	2.78 (1.93-4.02)	<.001	3.04 (1.86-4.98)	<.001
Stroke	1.47 (1.33-1.63)	<.001	1.65 (1.41-1.94)	<.001
Trauma	3.16 (2.93-3.41)	<.001	3.19 (2.86-3.57)	<.001

Abbreviations: NA, not applicable; OR, odds ratio.

^a^ Outcome variable is yes or no regarding acceptance. The primary exposure is boarding hours (quartiles). Covariables include age (continuous variable), gender, rural hospital, weekend, referring unit (emergency department, intensive care unit, or floor), and prioritized diagnosis category.

Our sensitivity analysis limited to only prioritized diagnoses demonstrated similar odds of urban vs rural acceptances for obstetrics, trauma, and stroke but greater odds of urban STEMI acceptances (eTable 3 in Supplement 1). Our sensitivity analysis limited to ED-based transfer requests demonstrated similar odds of acceptance across all prioritized diagnoses (eTable 4 in Supplement 1).

## Discussion

To our knowledge, this is the first cross-sectional study to model the association of a regional referral hospital census (ED boarding and inpatient capacity) with regional IHT acceptance. We found that conditions of greater ED boarding and inpatient census were highly correlated and significantly associated with refusals of IHT requests. As anticipated, we found that IHT requests were disproportionately declined from rural hospitals, potentially negatively impacting the care of patients requiring specialty services and procedures. Although prioritization of specific conditions is effective at reducing rural disparities in IHT acceptance, these represent less than 5% of IHT requests. Importantly, our study also found that urban IHT requests are very common and frequently declined. Thus, our study raises the grave concern that in the face of this ongoing capacity crises, the US health system will be further limited in its aim to assure equitable access to highest levels of medical care for all US individuals, regardless of geography.

Our research highlights the sustained hospital capacity crisis that persists despite resolution of the COVID-19 pandemic. In this study, the only academic tertiary hospital in a highly rural state decreased its usual acceptance rate of more than 70% to roughly 40%. This reduction in IHT acceptances occurred in the setting of an unprecedented and unsustainable rise in inpatient census, neither of which have normalized, and as the hospital attempted to gradually reduce inpatient volume, there was a sustained and rapid rise in ED boarding. This finding illustrates how capacity strained hospitals function as a closed system, as patient overload location simply shifts. This overload spills into the region, with severe ED boarding associated with lower IHT acceptance. While this is one hospital’s experience, nationally, academic medical centers are increasingly experiencing worsening ED boarding,^15^ and IHTs are reduced in regions with caseload strained hospitals.^8^

There is an extensive body of literature demonstrating ED boarding is associated with negative patient outcomes.^3,4^ It is associated with delays in definitive care, missed care, medication errors, delirium, higher morbidity and mortality, and longer hospital lengths of stay.^16,17^ It is also associated with poor patient experience, as well as physician burnout.^16^ To address these quality and safety harms, referral hospitals reduce IHT acceptances, prioritizing the care of its current patients and preserving access for the most complex IHTs. However, to the extent that referral hospitals serve as points of access to higher levels of expertise and services, this results in potential harms to regional patients. At the time of the study’s conclusion (spring 2023), 60% of transfers were declined; this places an enormous burden on physicians in community hospitals with limited resources, who must call multiple hospitals to find an accepting facility.^18^ This results in delays in transfer, as well as unintended harms to patients in the sending ED because patients who should be transferred, but cannot, consume a disproportionate number of resources. Additionally, because uninsured and Medicaid enrolled patients experience greater levels of IHT,^19^ inability to achieve transfer may cause worsening disparities. IHT, which can be considered a form of treatment, facilitates improved outcomes for life-threatening conditions, particularly for rural patients.^20^ In the absence or restriction of IHT availability for rural residents and those living more remotely from tertiary care centers, in many ways, geography becomes destiny.^21^

Previous efforts to reduce boarding at institutional levels have largely failed, because the solutions to ED boarding do not lie within the ED, but require systemic changes at the hospital, regional, and national levels.^1^ At the federal level, the Department of Health and Human Services convened an ED boarding task force to identify solutions.^22^ Such efforts may focus on load-leveling within hospital units and between hospitals regionally, expanding postacute care options (eg, skilled nursing), and supporting the nursing workforce to ensure all licensed beds are able to be staffed. While there is reasonable concern that the US health system has inadequate inpatient capacity,^23^ state-based interhospital transfer centers in Washington, Arizona, and Minnesota were effective during the COVID-19 pandemic at load-leveling hospitalizations regionally and improving health equity.^10,24^ These centers mirror the concepts described by the Office of the Assistant Secretary for Preparedness and Response Medical Operations Coordinating Centers, which can improve organization and coordination of transfer networks to optimize capacity distribution.^25,26^ Hospital-level solutions are likely less effective but could include telemedicine, which improves quality of care and may reduce transfers,^27,28,29^ or could deploy triage metrics and detailed data collection processes, allowing for data-driven decisions in IHT acceptance and ongoing quality improvement review. Ideally, there is a system of care within IHT that considers patient factors and sending and receiving hospital resources (eg, specialists) across all conditions.

### Limitations

This study has limitations. Although these data originate from a single center, the hospital is the primary referral center and sole level I trauma, comprehensive stroke, and high-risk obstetrics center in the state. While some IHTs cross state borders (eg, the southeastern portion of the New Mexico to Texas), most of the state’s transfers requiring the highest level of care come to the academic medical center studied, making it reasonable to consider this a statewide analysis. A second limitation is the lack of clinical or acuity measures, which might influence prioritization of transfer request acceptance. We sought to address this limitation through our sensitivity analyses, focused on prioritized conditions and ED-based transfers. It is likely that urban patients who require transfer are more complex, which is supported by higher percentages of urban transfer requests originating from a floor or ICU location. It is also likely that the threshold for IHT request for rural patients is lower than urban patients, and we considered that rural patients may be less sick at time of presentation. However, the existing literature suggests that rural patients in the US are less healthy,^30^ more likely to die from leading causes of death,^31^ and are more likely to die during a hospitalization.^32,33^ Third, we could not link transfers to electronic health records and could not examine patient outcomes. Fourth, there may be other measures of hospital operations not accounted for in the analysis, which may influence transfer acceptance decisions, including surgical volume, nurse staffing and bed closures, or various temporary hospital policies. We could not report on findings stratified by racial or ethnic classifications because the transfer center does not collect this information. Fifth, we could not report on insurance status because the transfer center specifically prohibits collecting any information regarding payment or insurance. However, given that the uninsured and Medicaid recipients have higher rates of IHT, it is likely insurance played a role in IHT decisions.

## Conclusions

In this cross-sectional study of statewide transfer request data, we found the severity of ED boarding and inpatient census was associated with refusals of IHT requests. While this disproportionately impacted rural patients, most refusals were for urban patients. Our findings raise complex ethical questions; notably, are chronically overburdened hospitals the victims or the perpetrators? Trapped in a dysfunctional health system, they face seemingly impossible decisions as they seek to fulfill their obligations to local and regional patients, and the resultant actions only perpetuate existing dysfunction and inequity.

## Notes

Supplement 1. **eTable 1.** Top 20 Diagnoses Accepted, in Order of Accepted Proportion **eTable 2.** Monthly IHT Acceptance Rate, Boarding Hours, Inpatient Census **eTable 3.** Sensitivity Analysis 1: Predicted Means and Odds of Acceptance, Comparing IHT Requests From Urban vs. Rural Hospitals, Across All Referring Units (e.g., ED, Floor, ICU) **eTable 4.** Sensitivity Analysis 2: Predicted Means and Odds of Acceptance, Comparing IHT Requests From Urban vs. Rural Hospitals, But Limited to Only IHT Requests From EDs

Supplement 2.
Data Sharing Statement
