# Supplementary material for: Emergency Department Boarding, Inpatient Census, and Interhospital Transfer Acceptances
Source: JAMA Netw Open. 2025 May 28;8(5):e2512299. doi: 10.1001/jamanetworkopen.2025.12299 (PMC12120653; doi:10.1001/jamanetworkopen.2025.12299)
Supplement: Supplement 1. — eTable 1. Top 20 Diagnoses Accepted, in Order of Accepted Proportion eTable 2. Monthly IHT Acceptance Rate, Boarding Hours, Inpatient Census eTable 3. Sensitivity Analysis 1: Predicted Means and Odds of Acceptance, Comparing IHT Requests From Urban vs. Rural Hospitals, Across All Referring Units (e.g., ED, Floor, ICU) eTable 4. Sensitivity Analysis 2: Predicted Means and Odds of Acceptance, Comparing IHT Requests From Urban vs. Rural Hospitals, But Limited to Only IHT Requests From EDs [file jamanetwopen-e2512299-s001.pdf]

Supplemental Online Content

Greenwood-Ericksen M, Kamdar N, Swenson K, et al. Emergency department boarding, inpatient census, and interhospital transfer acceptances. *JAMA Netw. Open*. 2025;8(5):e2512299. doi:10.1001/jamanetworkopen.2025.12299

eTable1. Top 20 Diagnoses Accepted, in Order of Accepted Proportion
eTable 2. Monthly IHT Acceptance Rate, Boarding Hours, Inpatient Census
eTable 3. Sensitivity Analysis 1: Predicted Means and Odds of Acceptance, Comparing IHT Requests From Urban vs. Rural Hospitals, Across All Referring Units (e.g., ED, Floor, ICU)
eTable 4. Sensitivity Analysis 2: Predicted Means and Odds of Acceptance, Comparing IHT Requests From Urban vs. Rural Hospitals, But Limited to Only IHT Requests From EDs

This supplemental material has been provided by the authors to give readers additional information about their work.

© 2025 Greenwood-Ericksen M et al. *JAMA Network Open*.

eTable1. Top 20 Diagnoses Accepted, in Order of Accepted Proportion.

Diagnoses	Accepted Proportion (%)
**Pre-Eclampsia**	**91.30**
**Ectopic Pregnancy**	**90.91**
**Stab Wound**	**88.46**
**MVA (Motor Vehicle Accident)**	**87.19**
**Trauma**	**86.64**
**Ovarian Torsion**	**86.36**
**GSW**	**86.23**
**Epidural Bleed**	**85.71**
Post Op Bleeding	85.71
**Pre-Term Labor**	**84.80**
**Ruptured Globe**	**84.38**
Aortic Occlusion	84.00
**Hypertension - Gestational**	**84.00**
**Assault**	**83.03**
**Prematurity <37 Weeks**	**82.86**
Airway Obstruction	82.14
**STEMI**	**81.12**
**Premature Rupture of Membranes**	**78.45**
Tracheostomy Complications	77.78
**Fall**	**77.76**

Legend. Accepted proportion was calculated for each diagnosis by dividing the number of accepted for X condition divided by total requests for X condition. **Bolded** diagnoses are considered prioritized conditions (stroke, STEMI, OB, trauma).

© 2025 Greenwood-Ericksen M et al. *JAMA Network Open*.

eTable 2. Monthly IHT Acceptance Rate, Boarding Hours, Inpatient Census.

Month	Year	IHT Acceptance Rate	Monthly boarding hours (average)	Monthly inpatient census (average)
**January**	2019	0.76	391.6	303
**February**	2019	0.74	382.9	318.75
**March**	2019	0.77	558.7	322
**April**	2019	0.76	523.5	325
**May**	2019	0.81	406.6	317
**June**	2019	0.76	527.6	325
**July**	2019	0.76	364.7	306.5
**August**	2019	0.78	371.4	318.75
**September**	2019	0.75	451.7	325
**October**	2019	0.74	573.6	331.25
**November**	2019	0.74	385.6	312.5
**December**	2019	0.75	498.6	318.75
**January**	2020	0.73	566.3	325
**February**	2020	0.74	493.4	318.75
**March**	2020	0.75	499.5	300
**April**	2020	0.81	72.7	237.5
**May**	2020	0.76	280.0	293
**June**	2020	0.73	543.2	347.5
**July**	2020	0.71	636.9	337.25
**August**	2020	0.72	683.9	335
**September**	2020	0.72	706.3	337.75
**October**	2020	0.68	583.4	337.75
**November**	2020	0.63	268.5	350
**December**	2020	0.69	239.5	361
**January**	2021	0.72	186.6	350
**February**	2021	0.72	218.6	353.5
**March**	2021	0.70	300.0	367
**April**	2021	0.74	382.8	367
**May**	2021	0.70	434.2	475.5
**June**	2021	0.69	500.1	495.6
**July**	2021	0.67	585.4	503.2
**August**	2021	0.56	500.9	489.6
**September**	2021	0.53	484.0	494

© 2025 Greenwood-Ericksen M et al. *JAMA Network Open*.

**October**	2021	0.52	609.7394	525.9
**November**	2021	0.53	594.8314	530.9
**December**	2021	0.45	638.2215	538
**January**	2022	0.33	698.3345	533.5
**February**	2022	0.42	578.0203	499.6
**March**	2022	0.49	586.4489	496.1
**April**	2022	0.56	713.3886	496.4
**May**	2022	0.48	879.5745	497
**June**	2022	0.51	792.9862	474.8
**July**	2022	0.53	750.0062	482.8
**August**	2022	0.48	547.7154	481.2
**September**	2022	0.50	731.605	468.5
**October**	2022	0.45	870.5099	452.9
**November**	2022	0.44	959.6066	492.4
**December**	2022	0.38	1096.08	490.7
**January**	2023	0.43	1194.109	477.1
**February**	2023	0.42	1251.557	478.5
**March**	2023	0.41	1257.402	466.1
**April**	2023	0.41	1298.808	471
**May**	2023	0.41	1277	453

Legend. Source of Figure 2 data and associated correlations. Daily ED boarding hours were averaged across a month (1st -30th or 31st) and inpatient monthly averages were provided by hospital administration.

© 2025 Greenwood-Ericksen M et al. *JAMA Network Open*.

eTable 3. Sensitivity Analysis 1: Predicted Means and Odds of Acceptance, Comparing IHT Requests From Urban vs. Rural Hospitals, Across All Referring Units (e.g., ED, Floor, ICU).

**All IHT Requests**	**Urban**	**Rural**	**Odds Ratio (Rural vs. Urban)** Ref: urban
**Diagnosis Category**	**Predicted Mean**	**Predicted Mean**	
**OB**	0.86 (0.83, 0.89)	0.82 (0.75, 0.87)	0.72 (0.46,1.12)
**STEMI**	0.84 (0.77, 0.89)	0.63 (0.42, 0.81)	0.33 (0.12, 0.88)
**Stroke**	0.65 (0.62, 0.68)	0.59 (0.52, 0.65)	0.77 (0.57, 1.04)
**Trauma**	0.76 (0.74, 0.78)	0.76 (0.71, 0.79)	0.97 (0.80, 1.19)
**All Others**	0.54 (0.53, 0.56)	0.45 (0.43, 0.47)	0.68 (0.62, 0.74)

Legend: These predicted means compare the odds of a prioritized condition (e.g., OB, STEMI, Stroke, Trauma) being accepted from an urban vs. rural hospital. IHT interhospital transfer; OB obstetric; STEMI ST-elevation myocardial infarction. Predicted means are the predicted population margins and represent the estimates of the marginal means over a balanced population.

© 2025 Greenwood-Ericksen M et al. *JAMA Network Open*.

eTable 4. Sensitivity Analysis 2: Predicted Means and Odds of Acceptance, Comparing IHT Requests From Urban vs. Rural Hospitals, But Limited to Only IHT Requests From EDs

**Only ED-originating IHT requests**	**Urban**	**Rural**	**Odds Ratio (Rural vs. Urban)** Ref: urban
**Diagnosis Category**	**Predicted Mean**	**Predicted Mean**	
**OB**	0.87 (0.81, 0.92)	0.82 (0.69, 0.90)	0.66 (0.28, 1.55)
**STEMI**	0.88 (0.81, 0.92)	0.72 (0.49, 0.87)	0.37 (0.12, 1.09)
**Stroke**	0.72 (0.69, 0.74)	0.66 (0.59, 0.72)	0.77 (0.56, 1.06)
**Trauma**	0.82 (0.80, 0.83)	0.81 (0.78, 0.84)	0.95 (0.78, 1.16)
**All Others**	0.61 (0.60, 0.62)	0.52 (0.50, 0.55)	0.69 (0.63, 0.76)

Legend: IHT interhospital transfer; OB obstetric; STEMI ST-elevation myocardial infarction. Predicted means are the predicted population margins and represent the estimates of the marginal means over a balanced population.

© 2025 Greenwood-Ericksen M et al. *JAMA Network Open*.
