# Supplementary material for: Emergency Department Boarding, Inpatient Census, and Interhospital Transfer Acceptances
Source: JAMA Netw Open. 2025 May 28;8(5):e2512299. doi: 10.1001/jamanetworkopen.2025.12299 (PMC12120653; doi:10.1001/jamanetworkopen.2025.12299)
Supplement: Supplement 2. — Data Sharing Statement [file jamanetwopen-e2512299-s002.pdf]

Data Sharing Statement

Greenwood-Ericksen. A Statewide Analysis of Emergency Department Boarding and Interhospital Transfer Acceptances. *JAMA Netw Open*. Published May 28, 2025. doi:10.1001/jamanetworkopen.2025.12299

Data
Data available: No
